# Physicochemical and Techno-Functional Properties of Dried and Defatted Porcine Liver

**DOI:** 10.3390/biom12070926

**Published:** 2022-07-01

**Authors:** Blanca Abril, Eduardo A. Sánchez-Torres, Mònica Toldrà, Jose Benedito, Jose V. García-Pérez

**Affiliations:** 1 Department of Food Technology, Universitat Politècnica de València, UPV, Camí de Vera, s/n, 46022 Valencia, Spain; blaabgis@alumni.upv.es (B.A.); edsantor@tal.upv.es (E.A.S.-T.); jjbenedi@tal.upv.es (J.B.); 2 Institute of Food and Agricultural Technology (INTEA), XIA (Catalonian Network on Food Innovation), Escola Politècnica Superior, University of Girona, C/Maria Aurèlia Capmany 61, 17003 Girona, Spain; monica.toldra@udg.edu

**Keywords:** porcine liver, liver protein, drying, defatting, physicochemical characteristics, techno-functional properties

## Abstract

Porcine liver has a high nutritional value and is rich in proteins, minerals, and vitamins, making it an interesting co-product to alleviate the growing global demand for protein. The objective of this study was to analyze how the drying and defatting processes of porcine liver affect the physicochemical and techno-functional properties of its proteins. Two drying temperatures (40 and 70 °C) were studied, and dried samples were defatted using organic solvents. The drying process turned out to be an effective method for the stabilization of the protein fraction; however, when the drying temperature was high (70 °C), greater protein degradation was found compared to drying at a moderate temperature (40 °C). Regarding the defatting stage, it contributed to an improvement in certain techno-functional properties of the liver proteins, such as the foaming capacity (the average of the dried and defatted samples was 397% higher than the dried samples), with the degree of foaming stability in the liver dried at 40 °C and defatted being the highest (13.76 min). Moreover, the emulsifying capacity of the different treatments was not found to vary significantly (*p* > 0.05). Therefore, the conditions of the drying and defatting processes conducted prior to the extraction of liver proteins must be properly adjusted to maximize the stability, quality, and techno-functional properties of the proteins.

## 1. Introduction

In recent years, the consumption of pork-derived food products has been on the rise, which has stimulated its production in Europe. The main change that has taken place in the pork sector consisted of the transition from a multifunctional livestock, typical of organic-based livestock activity, to an intensive livestock model [1]. The more intensive production model is having a negative impact on the environment both in terms of the use of resources (nitrogen, phosphorus, land, and water) and greenhouse gas emissions [2]. Furthermore, a large number of co-products and a great amount of animal waste are generated in the meat industry, usually in slaughterhouses [3], which also increases its environmental impact. In this way, if we consider both economic and environmental issues, any action whose purpose is to revalue meat co-products is of great interest for this industry. In addition, the recovery and revalorization of co-products is a current demand of our society.

The valorization of meat co-products depends on several factors, such as the country, culture, religion, economy, consumer preferences, etc. [4]. In general, hearts, tongues, livers, lungs, and kidneys from animals such as pigs, cows, and lambs, have traditionally been consumed as food or as food ingredients in many countries [5]. However, Llauger et al. [6] conducted a study in Spain on the attitude of consumers towards the development of meat products from animal co-products and offal. The results reflected how society has a great interest in contributing to the environment by means of the valorization of these co-products, but their typical flavor hinders their acceptability and consumption despite being considered healthy.

In general, the last few decades have witnessed a drop in the use of animal co-products for human consumption [7]. Most co-product consumption is linked to their use as an ingredient in the manufacturing of traditional meat products, such as pâté (from the liver), blood sausages, or animal feed [8]. In addition, meat co-products have been used in the development of new functional ingredients [9], such as bioactive peptides obtained from the proteolysis mechanisms of endogenous meat enzymes (calpains) or exogenous plant enzymes (papain, bromelain, or ficin) [10], and antimicrobial agents [11]. Co-products have also been used to produce biodiesel [12]. Moreover, the growth of world population and the increase in demand for food, combined with actions in favor of the sustainability of the meat industry, have highlighted the need to find alternative protein sources (pulses, cereals, insects, fungi, algae, and protein recovered from meat or fish by-products) of nutritional and sensory quality, with a milder environmental impact [5]. In this regard, the nutritional value of meat co-products is closely comparable to lean meat [13], and they are sources of high-quality proteins with excellent techno-functional properties.

The use of proteins from meat co-products has attracted interest as a result of their use in human nutrition [14]. Protein hydrolysates can have various applications as food ingredients, due to their emulsifying properties or foaming agents [15]. These proteins may also be applied in nutritional formulas for special adult diets, in extra protein supplements for the elderly, in formulas for infants with allergies to intact food proteins or with congenital metabolic disorders, and as nutraceuticals [16].

Porcine liver is an interesting co-product of the meat industry from a nutritional point of view, as it is rich in protein (22.05 ± 1.38%), low in fat (2.94 ± 0.50%), and contains minerals and nutrients, such as essential amino acids and fatty acids [17]. However, liver has 71.59 ± 3.42% moisture; therefore, in the same way as meat and other offal, it is a perishable product and has a limited shelf life [18]. Therefore, its previous dehydration is convenient in order to prolong its shelf life and facilitate its handling and storage before further use in applications, such as protein extraction [19]. Moreover, the removal of the fat fraction would also facilitate the recovery of the protein fraction, and would limit degradation reactions, such as lipid oxidation and rancidity. However, it is important to investigate how water and fat reduction affects protein quality and functionality. Proteins have functional properties of great technological interest, such as their capacity to solubilize, gel, foam, or emulsify, which makes them useful for the purposes of improving the creation of new products derived from meat co-products, as well as for reducing shear strength and improving the texture of meat products [20].

Drying and defatting operations have been used for the isolation and subsequent extraction of proteins, mainly in vegetables such as lupine [21], or from insect flour, in order to make meat analogues [22]. No references have been found to the effect of drying and defatting on liver protein quality. Depending on the matrix from which the protein fraction is extracted, the drying temperature is critical, directly affecting protein stability and, therefore, its techno-functional properties, such as solubility and foaming capacity. Thus, for example, temperatures above 80 °C negatively affected the solubility and foaming capacity and stability of lupine protein [21]. However, the defatting operation can cause changes in the amino acid composition and functionality of the proteins, such as an increase in the foaming and emulsifying capacity, as well as an increase in the quality of the insect protein in terms of essential amino acids [23]. Thus, the main objective of this study was to evaluate the effect of the drying and defatting processes on the physicochemical and techno-functional properties of porcine liver.

## 2. Materials and Methods

### 2.1. Raw Material and Sample Preparation

Raw porcine livers from an industrial slaughterhouse were transported to the laboratory at 4 °C. Liver conditioning consisted of (i) the separation of its four main lobes, (ii) the splitting of each lobe into two parts, (iii) vacuum packaging (200 × 300 PA/PE, Sacoliva, Barcelona, Spain), and (iv) freezing (at −20 °C) until processing.

#### 2.1.1. Drying Process

Before drying, vacuum packaged samples were tempered at 2 °C for 2 h in order to facilitate further handling. Using a household device, cylinders of standardized dimensions (12.6 mm diameter × 15 mm height) were obtained. In each drying run, eight cylinders, with a total weight of approximately 15 g, were used.

The drying experiments were carried out at moderate-low (40 °C) and moderate-high (70 °C) temperatures using a convective oven (FD 56, Binder GmbH, Tuttlingen, Germany) with an air speed of 1.3 m·s^−1^. Then, eight cylindrical porcine liver samples were placed in a porcelain crucible and their weight was recorded manually every 15 min. The finalization criterion was set at a weight loss of 70% of the initial weight. At the end of the drying tests, the dried porcine liver samples were ground, vacuum packed in 200 × 300 PA/PE bags (Sacoliva, Barcelona, Spain), and stored under refrigeration at 4 °C until their physicochemical and techno-functional characterization. The drying experiments were replicated three times at both temperatures. Subsequently, the porcine liver replicates dried at the same temperature (D-40°C or D-70°C) were ground and mixed, with the aim of obtaining a representative sample of each drying experiment and, thus, performing the analyses of the physicochemical and techno-functional properties of dried porcine liver.

#### 2.1.2. Defatting Process

For liver defatting purposes, standard method 991.36 [24] was used, based on the use of Soxhlet equipment and an organic solvent. Thus, 3 g of dried and ground porcine liver were weighed. Then, samples were placed in a filter paper cartridge (porous material). Subsequently, the sample was placed in the chamber of the Soxhlet extractor, which consisted of a balloon-flask (previously desiccated for 1 h at 125 °C, to eliminate ambient humidity), the chamber of the Soxhlet extractor (where the cartridge was inserted), a condenser, and a water bath at a temperature above 70 °C (boiling point of the organic solvent used). In each extractor, 75 mL of organic solvent, petroleum ether (C_6_H_6_), was used. Thus, the heated solvent, located in the flask-balloon immersed in the bath at 70 °C, evaporated on reaching the condenser and fell on the sample cartridge, extracting the fat. The condensed solvent returned to the boiling flask and the process was run continuously for 5 h. Afterwards, the balloons containing the fat dissolved in the organic solvent were rota-evaporated to separate the solvent from the fat. Meanwhile, in order to remove the solvent in the dried liver, the cartridges were placed in a vacuum oven at 70 °C for 4 h. Defatting experiments were replicated three times at both drying temperatures (40 and 70 °C). Subsequently, the dried and defatted porcine liver was removed from the cartridge, and then the replicates (DD-40 °C and DD-70 °C) were ground and mixed with the aim of obtaining representative samples to perform the physicochemical and techno-functional analyses. The samples were vacuum packed in PA/PE bags and stored under refrigeration at 4 °C until characterization.

### 2.2. Modelling of Air-Drying Kinetics

For the mathematical description of the drying kinetics at 40 °C and 70 °C, the Weibull empirical model was used [25]. The Weibull model is based on Equation (1).

(1) $$W_{t}=W_{e}+\left(W_{0}-W_{e}\right)\cdot \exp{\left[-\left(\frac{t}{\beta }\right)\right]}^{\alpha }$$

where *W_t_* is the moisture content (kg water/kg dry matter) at time *t* (s), *W_e_* is the equilibrium moisture content (kg water/kg dry matter), *W*_0_ is the initial moisture content (kg water/kg dry matter), and *β* (s) and *α* are the kinetic and shape parameters of the model, respectively. *W_e_* was estimated from the equilibrium data reported by Sanchez-Torres et al. [19].

When the value of *α* is equal to 1, the model corresponds to first order kinetics, with a constant water loss rate. When *α* > 1, the reaction rate for the Weibull model is increasing as a function of time and when *α* < 1, it is decreasing [26]. The Weibull model (Equation (1)) was fitted to the experimental data and the kinetic and shape parameters were determined. The identification of Weibull parameters (*α* and *β*) was carried out using an optimization procedure that minimized the sum of the squared differences between the experimental and calculated average moisture contents of the samples. For that purpose, the non-linear optimization algorithm of the Generalized Reduced Gradient (GRG), available in a Microsoft Excel^TM^ spreadsheet from MS Office 2019, was used [27]. The percentages of mean squared error (%MRE, Equation (2)) and explained variance (%VAR, Equation (3)) were determined to evaluate the model’s goodness of fit.

(2) $$\;\%\mathrm{MRE}=\frac{100}{N}\left[\sum_{i=1}^{N}\frac{\left|\mathrm{Wexp}-\mathrm{Wcal}\right|}{\mathrm{Wexp}}\right]$$

(3) $$\%\mathrm{VAR}=\left[1-\frac{{\mathrm{Sxy}}^{2}}{{\mathrm{Sy}}^{2}}\right]100$$

where *W_exp_* and *W_cal_* are the experimental and the estimated moistures, respectively; *N* is the number of experimental data, and *S_xy_* and *S_y_* are the standard deviations of the estimation and the sample deviation, respectively.

### 2.3. Physicochemical Characteristics

#### 2.3.1. Proximate Analysis

Standard methods were used to analyze the proximate composition of dried porcine liver. Each sample was analyzed in triplicate. Moisture, ash, protein, and fat contents were determined by following the procedures established by the Association of Official Analytical Chemists [24]. Moisture and ash contents were determined gravimetrically using a hot air oven and a muffle furnace, respectively. The protein content was estimated from the total Kjeldahl nitrogen (TKN × 6.25) by using a Gerhardt KB20 digestion system (Gerhardt GmbH & Co. KG, Königswinter, Germany) and a Büchi K-314 distillation unit (Büchi Labortechnik AG, Flawil, Switzerland). The total fat content was determined gravimetrically by Sohxlet extraction with diethyl ether.

#### 2.3.2. Color Parameters

The color of dried liver samples was measured by determining the CIE L*, a* and b* color parameters, with L* representing the lightness on a scale of 0 (dark) to 100 (white); a* the redness-greenness value; and b* the yellowness-blueness value, using a Minolta Chroma Meter CR-300 with a CR-A33f glass light projection tube (Minolta Co, Ltd., Osaka, Japan). The measurements were taken using diffuse illumination, a D65 light source, and 2° standard observer. The colorimeter was calibrated using a standard white ceramic plate (L* = 97.15, a* = −5.28 and b* = +7.82). The color of each sample was measured in triplicate. The angular coordinates of chroma (C*) and hue angle (H°) were calculated according to Equations (4) and (5), respectively.

(4) $$C*=\sqrt{{(a*}^{2}{+b*}^{2})}$$

(5) $$H^{o}=\tan^{-1}\left(\frac{b*}{a*}\right)$$

#### 2.3.3. Differential Scanning Calorimetry (DSC) Analysis

Differential Scanning Calorimetry (DSC) analyses of samples were performed in a DSC (Q 2000 calorimeter, TA Instruments, New Castle, DE, USA), under a heating program from 20 to 100 °C and at a heating rate of 3 °C·min^−1^. Solutions of reconstituted dried liver samples with the same percentage of protein (*w*/*v*) as raw porcine liver (19.20%) were analyzed. From the DSC thermograms (heat flow curve as a function of temperature), the area of the endothermic transition peaks was calculated by integration and, by the use of a straight baseline, the variation in the global transition enthalpy of the protein denaturation (∆H, J/g) was also calculated. The initial temperature of the endothermic peak (*T_i_*) and the denaturation temperatures (*T_d_*_1_ and *T_d_*_2_) were also obtained, considering that they correspond to the temperatures at which the minimum of the two main endothermic peaks of denaturation were recorded in the thermograms. TA Instruments’ Universal Analysis software was used.

### 2.4. Techno-Functional Properties

#### 2.4.1. Protein Solubility

The protein solubility of dried porcine liver samples was analyzed using the method described by Morr et al. [28], with slight modifications. First, 1 g of ground dried liver samples was diluted in distilled water (100 mL). The solutions were stirred for 30 min on a magnetic stirring plate, thus avoiding vortex formation. After stirring, aliquots of the solutions were centrifuged at 20,000× *g* for 30 min at 20 °C (Sorvall RC-SC plus, Dupont Co., Newton, CT, USA) and decanted. The protein solubility was calculated as the percentage of soluble protein content in the supernatant relative to the total protein content of dried liver samples. The protein content in both was determined by the Kjeldahl method AOAC 954.01 [24]. Each determination was carried out in duplicate for each drying condition.

#### 2.4.2. Foaming Properties

The foaming properties were determined as described in Toldrà et al. [29]. Three aliquots of 200 mL of dried liver protein solutions (5 g/L) from each sample were prepared in distilled water, and then transferred to 1000 mL volumetric flasks. The solutions were whipped in a mixer (Multimix M700, Braun Española S.A., Esplugues de Llobregat, Barcelona, Spain) with two whisks (Ø = 5 cm) at 1000 rpm for 10 min. The flasks were placed on a rotational plate during mixing to form homogeneous foams. Afterwards, the foaming capacity (FC) was determined as the volume (mL) of foam after 2 min at rest. The foam stability was determined using a gravimetric method as follows: measured quantities of foam were carefully placed in three dry stainless-steel sieves to let the released liquid drain, and the remaining foam was weighed every 10 min for a period of 60 min. The percentage of remaining foam versus time was plotted, and relative foam stability (RFS), defined as the time (min) needed for the disappearance of 50% of the initial foam, was calculated by fitting an exponential decay function to the experimental data using Equation (6).

(6) $${y=B}_{0}\cdot e^{\left({-B}_{1}\times t\right)}$$

where *B*_0_ is the initial foam percentage, *t* is the time (min), and *B*_1_ the foam disappearance rate (min^−1^). The measurements were taken in triplicate.

#### 2.4.3. Emulsifying Properties

The emulsifying properties were determined following the turbidimetric method described by Pearce and Kinsella [30] and slightly modified by Parés and Ledward [31]. The solutions of dried liver samples were prepared at 5 g/L of protein (*w*/*v*). Then, 150 mL of liver protein solution was homogenized along with 50 mL of commercial corn oil using a hand-operated laboratory piston-type homogenizer (MFC MicrofluidizerTM Series 5000, Microfluidics Corporation, Newton, MA, USA) at 12 MPa, giving 40 L/h output flow for 90 s, with recirculation. The temperature was maintained at 20 °C. The preparations were carried out in triplicate for each sample. The emulsions were diluted 2500-fold with 0.1% sodium dodecyl sulphate (SDS), immediately after homogenization (t = 0) and after 10 min of emulsion rest (t = 10). The absorbance of the diluted emulsions was then determined at 500 nm in a spectrophotometer (CE 7400, Cecil Instruments Ltd., Cambridge, UK). Each determination was performed in duplicate. The results were reported as the Emulsifying Activity Index (EAI) and the Emulsion Stability Index (ESI). The EAI (m^2^ g^−1^ protein) and ESI (min) were calculated by means of Equations (7) and (8), respectively.

(7) $$\mathrm{EAI}=\frac{2\cdot T}{\phi \cdot C}$$

(8) $$\mathrm{ESI}=\frac{T\times \Delta t}{\Delta T}$$

where *T* is the turbidity, ϕ is the volume fraction of the dispersed phase (calculated as the volume of the oil phase divided by the total volume of the emulsion), *C* is the weight of protein per unit volume of aqueous phase before the emulsion is formed (g/mL), and Δ*T* is the change in turbidity (*T*) occurring during Δ*t* (10 min). The EAI has units of the area of stabilized interface per unit weight of protein.

### 2.5. Statistical Analysis

To evaluate the significance of the differences identified between the Weibull parameters, an analysis of variance (ANOVA) was performed and the LSD (least significant differences) intervals were identified. Likewise, from the ANOVA and the LSD intervals, the influence of the drying temperature and the drying and subsequent defatting process on the physicochemical and techno-functional characteristics was analyzed with a 95% confidence level (*p* < 0.05). A statistical analysis was performed using Centurion XVI software (Statpoint Technologies Inc., Warrenton, VA, USA).

## 3. Results

### 3.1. Modelling of Porcine Liver Drying Kinetics

**Figure 1:**
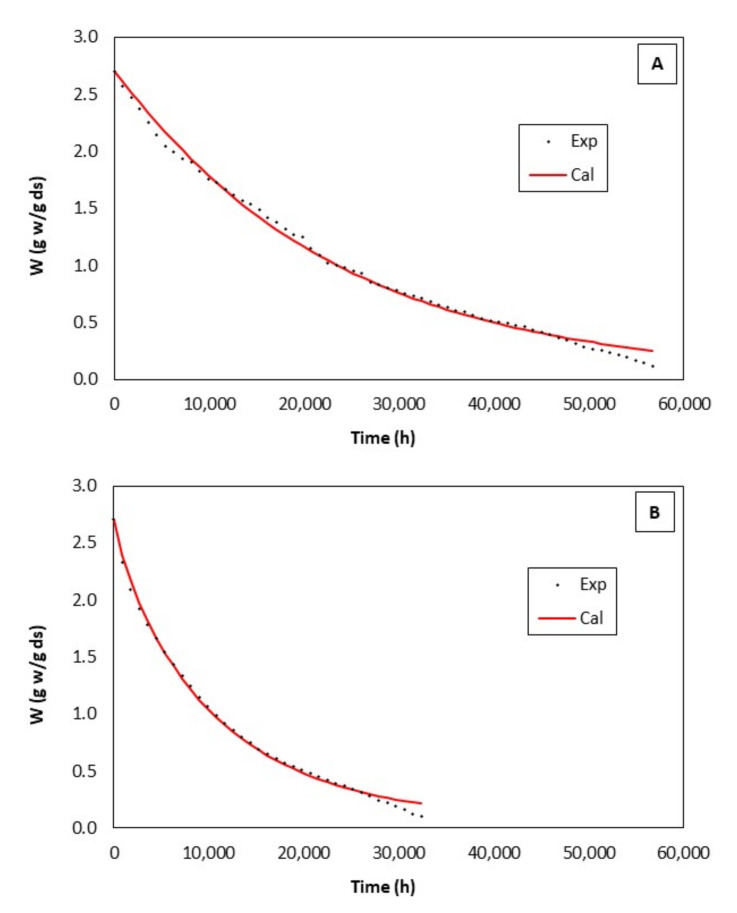
Experimental drying kinetics of porcine liver at 40 °C (**A**) and 70 °C (**B**) and the same drying kinetics calculated using the Weibull model.

The experimental drying kinetics of porcine liver at 40 °C and at 70 °C are shown in Figure 1. The moisture content varied between 2.704 and 0.115 (g water/g dry matter) for the liver dried at 40 °C and between 2.704 and 0.105 (g water/g dry matter) for the liver dried at 70 °C. Moreover, Figure 1 shows how the drying rate increased when the temperature rose (70 °C). Thus, in the drying kinetics at 40 °C, it took 56,700 s to reach a moisture of 0.1 g water/g dry matter, while at 70 °C, the time was shortened to 32,400 s. Therefore, a 42.9% reduction in the drying time was manifested at 70 °C.

The Weibull model provided a good description of drying kinetics, as shown in Figure 1A,B for the kinetics of dried porcine liver at 40 °C and 70 °C, respectively. The identified Weibull model parameters and goodness of fit estimators (%VAR and %MRE) are shown in Table 1. The percentages of explained variance obtained were high (99.5 ± 0.1 and 99.6 ± 0.1%, for 40 and 70 °C, respectively), and the MRE were equal to or lower than 10% (9.36 ± 0.33% and 10.14 ± 0.94%, for 40 and 70 °C, respectively), which indicates a reasonably satisfactory fit of the model (Table 1). On the one hand, as happened with the α parameter, it was equal to 1 for the drying kinetics at 40 °C (1.02 ± 0.03), which indicates that the drying rate follows a first order kinetic pattern. In the case of the drying kinetics at 70 °C, meanwhile, it was lower than 1 (0.86 ± 0.02), which indicated that the reaction rate was a decreasing function of time. On the other hand, the values of the β parameter were lower when drying porcine liver at 70 °C (1.1± 0.4 × 10^4^ s) than they were (2.3 ± 0.4 × 10^4^ s) for the drying kinetics of porcine liver at 40 °C. As the β parameter is inversely proportional to the drying velocity, a reduction in this value indicates an increase in velocity [25]; that is, drying velocity in the case of the kinetics at 70 °C is 54.17% higher than at 40 °C (Figure 1).

### 3.2. Physicochemical Characterisation 

#### 3.2.1. Chemical Composition

The chemical composition (moisture, protein, fat, and ash contents) of porcine liver samples is presented in Table 2. In general terms, dried samples presented a final moisture content of close to 10% (Table 2), which is expected considering a moisture loss of 70% during drying and an initial moisture content of nearly 73% [19]. The observed differences between the moisture contents of the samples dried at 40 and 70 °C were related to minor changes in the weight loss during drying and the natural variability in the dried porcine liver. Defatting treatments also contributed to a reduction in the moisture content of the samples. The moisture contents of the dried and subsequently defatted samples were 8.60 ± 0.22% and 6.62 ± 0.31%, in DD-40 °C and DD-70 °C, respectively, which are lower values than those found for the samples that were only dried (11.24 ± 0.40% in D-40 °C and 7.86 ± 0.27% in D-70 °C). This difference was linked to the fact that the Soxhlet defatting method occurs at a high temperature (~70 °C) and, therefore, a greater dehydration of the samples could be expected.

The defatting process enriched the protein content from around 16.5 to 22.6 % in the liver powders. A higher protein content was observed in the dried samples subjected to a defatting process (83.67 ± 1.15% and 82.98 ± 1.12% protein, in DD-40 °C and DD-70 °C, respectively), than in those that were only dried (61.10 ± 1.00% and 66.53 ± 0.06% protein in D-40 °C and D-70 °C, respectively).

Finally, the fat content in porcine liver samples dried at 40 °C and 70 °C was close to 20% at both temperatures, but slightly higher in samples dried at 40 °C (22.70 ± 1.57% fat in D-40 °C and 19.60 ± 0.55% fat in D-70 °C). Defatting reduced the fat content by 85.2% for the DD-40 °C (3.36 ± 0.96% fat) and 74.2% for the DD-70 °C (5.05 ± 0.05% fat).

#### 3.2.2. CIE L*a*b* Color Parameters

The color modifications caused by drying and defatting treatments to porcine liver were analyzed. On the one hand, the drying temperature did not lead to significant differences (*p* > 0.05) in the chroma (C*) and yellowness from CIE L*a*b* color parameters. However, the drying temperature had a significant effect (*p* < 0.05) on lightness, redness, and hue (H°) (Table 3). On the other hand, it is worth noting that defatting induced noticeable modifications in the color compared to those samples that were only dried, causing significant differences (*p* < 0.05) in the different CIEL*a*b* color parameters. As seen in Table 3, the removal of fat caused an increase in luminosity (L*), obtaining values of 73.95 ± 0.48 in DD-40 °C and 67.99 ± 0.53 in DD-70 °C, compared to 50.49 ± 0.52 in D-40 °C and 53.95 ± 1.37 in D-70 °C, and a decrease in the color coordinate (a*) (4.39 ± 0.08 in DD-40 °C and 4.97 ± 0.09 in DD-70 °C, compared to 8.83 ± 0.48 in D-40 °C and 7.09 ± 0.29 in D-70 °C). These changes were manifested in a tendency towards lightening (decrease in chroma (C*) and increase in hue (H°)). In this regard, the C* values obtained for the dried and defatted samples were 16.23 ± 0.04 in DD-40 °C and 17.66 ± 0.03 in DD-70 °C and the H° values were 74.97 ± 0.31 in DD-40 °C and 73.66 ± 0.28 in DD-70 °C. Regarding the dried samples, the C* values were 19.60 ± 0.59 in D-40 °C and 18.47 ± 0.61 in D-70 °C and the H° were 63.25 ± 0.91 in D-40 °C and 67.44 ± 0.28 in D-70 °C.

#### 3.2.3. Differential Scanning Calorimetry Analysis

**Figure 2:**
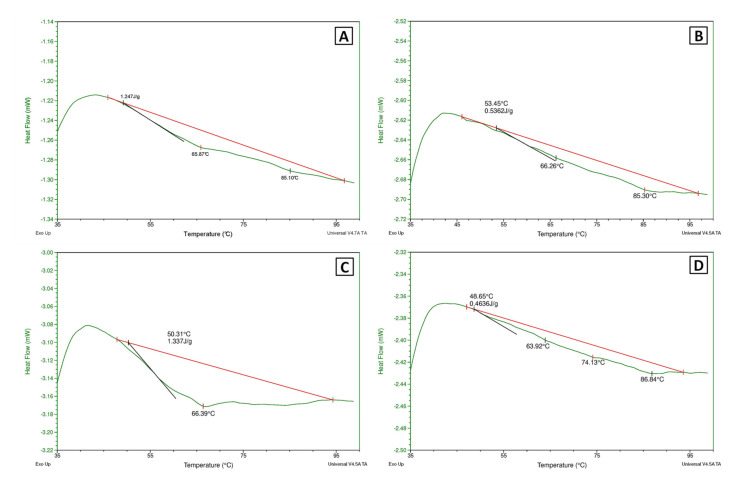
DSC thermograms of porcine liver samples dried at 40 °C (**A**) and 70 °C (**B**), and subsequently defatted (**C**,**D**) for samples dried at 40 and 70 °C, respectively, and reconstituted at 19.2% protein (*w*/*v*), as raw porcine liver. Endothermic heat flow (green lines); initial temperature (Tin) at which heat flow is detected (black lines), and baseline (red lines) for calculating the total Enthalpy of denaturation (ΔH).

The thermograms obtained by DSC are shown in Figure 2, and they graphically represent the variations in total enthalpy and the maximum denaturation temperature. According to [32], the process of the irreversible thermal protein denaturation of different meat proteins could take place in the temperature range of 45 °C to 90 °C. Table 4 shows the calorimetric parameters obtained by DSC from samples of porcine liver dried at 40 °C and 70 °C, with and without subsequent defatting. In general terms, dried and defatted samples presented lower enthalpy values than raw porcine liver (Table 4). This may reflect the structural modification in the protein matrix caused by drying and defatting. Moreover, differences in the endothermic heat flux were also found for dried samples (Figure 2). Drying at 40 °C resulted in a higher endothermic heat flux, which can be explained by considering the endothermic nature of the protein denaturation process. The samples dried at 70 °C have undergone greater irreversible denaturation leading to a lower total heat flux value during the calorimetric test. However, the liver dried at 40 °C presented a higher heat flux, because denaturation was milder than at 70 °C. In addition, it is important to highlight that defatting did not cause noticeable changes in the degree of protein denaturation, since similar values were obtained in the total heat flux between the dried and subsequently defatted samples and those only dried (1.25 J/g in D-40 °C vs. 1.34 J/g in DD-40 °C and 0.54 J/g in D-70 °C vs. 0.46 J/g in DD-70 °C).

### 3.3. Techno-Functional Properties

#### 3.3.1. Protein Solubility

The variation in the protein solubility of the different samples is a phenomenon that could be linked to the thermal denaturation produced by the drying and defatting treatments used. Thus, the drying temperature had a marked influence on the solubility of the proteins due to the fact that the samples dried at 70 °C were less soluble than those dried at 40 °C (Table 5). The variation in solubility is also explained by the results of the calorimetric analysis by DSC since, as commented on in Section 3.2.3, a lower heat flux (H_total_) is obtained in the liver dried at 70 °C than those obtained at 40 °C and when using raw liver, which indicates that drying at high temperatures causes a greater structural modification of the proteins and, therefore, an irreversible protein denaturation. Sánchez-Torres et al. [33] observed in porcine liver that the higher the drying temperature the lower the protein solubility of the dried material. Thus, Sánchez-Torres et al. [33] reported similar figures of the solubility than in this work (25.20% at 70 °C and 40.45% at 40 °C).

Defatted samples presented a slight reduction in protein solubility compared to those samples that were only dried, but the differences were not significant (*p* > 0.05), (45.74 ± 0.50% in D-40 °C vs. 42.60 ± 2.91% in DD-40 °C and 18.69 ± 0.46% in D-70 °C vs. 17.64 ± 0.30% in DD-70 °C). The addition of the solvent (ethyl ether) used for defatting caused the appearance of hydrophobic regions on the surface of the proteins [34] that could slightly affect the protein solubility.

#### 3.3.2. Surface Functional Properties (Foaming and Emulsifying)

**Figure 3:**
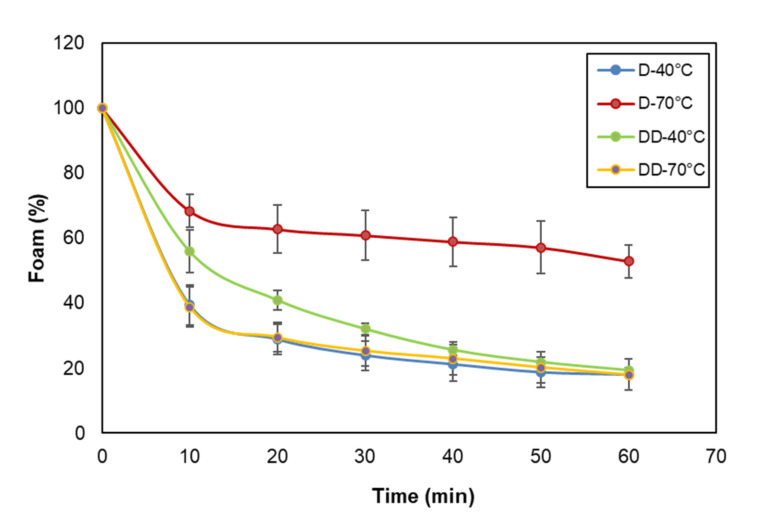
Foam stability (relative percentage of foam) of porcine liver protein solutions (0.5% *w*/*v*) of samples dried (D) at 40 and 70 °C and subsequently defatted (DD) (means ± SD, n = 3).

The foaming and emulsifying properties of dried and defatted porcine liver are shown in Table 6. The porcine liver dried at 40 °C and subsequently defatted (DD-40 °C) was the one that presented the highest foaming capacity together with a high degree of stability compared to the rest of the treatments (Foam capacity: 700.31 ± 32.35 mL and RFS: 13.76 min). These results were consistent with the effects of liver processing treatments on protein solubility. The drying temperature affected the protein solubility of porcine liver; consequently, the foaming properties were reduced, and there was a sharp drop in the % of foam formation at 20 min for every treatment, the foam formed being under 60% (Figure 3). Moreover, the defatting treatment contributed to the enhancement of the foaming properties of the protein fraction. This increase could be linked to the aforementioned effect provoked by the solvent used in defatting (ethyl ether) on the appearance of hydrophobic regions on the surface of the proteins, which enhances the surface-active properties of the proteins between the continuous and dispersed phases.

Although there were no variations observed between the emulsifying activity of the different treatments, a similar pattern to that found in the foaming properties was reproduced for the emulsifying stability. The ESI values of porcine liver fell as the drying temperature rose, whereas the defatting process did not contribute to an improvement in the emulsifying properties. However, when comparing the parameters of the functional surface properties obtained in the samples analyzed in the present study with those of protein extracts from porcine hearts and spleens at pH 6.5 (Table 6), derived from other research [29,35,36,37], it can be seen that all the samples analyzed have very poor surface functional properties, regardless of the treatment applied. However, the protein extracts from porcine heart and spleen did not undergo a prior drying or a drying and subsequent defatting process. Therefore, the functional properties of the proteins cannot be compared because the porcine liver proteins of the present study have undergone a structural modification brought about by denaturation as shown by the DSC and solubility results. For this reason, there are fewer functional properties of dried or dried/defatted porcine livers compared to protein extracts from raw porcine heart and spleen. 

## 4. Conclusions

The drying temperature influenced the physicochemical and techno- functional parameters of the porcine livers, 40 °C being the temperature that presented less protein degradation compared to the porcine liver dried at 70 °C. At the same time, the defatting stage contributed to an enhancement of certain techno-functional characteristics, such as foaming capacity and stability. Therefore, the treatments applied to the liver protein fractions should be determined according to the subsequent use of the liver ingredients. Further research should also investigate the impact of the drying process at milder temperatures (10, 20, and 30 °C) on the porcine liver in order to develop optimal thermal processing conditions that minimize the loss of quality and functionality in the protein matrix, while prolonging the shelf life of this highly perishable co-product.

## Figures and Tables

**Table 1:** Weibull model parameters for porcine liver dried at 40 °C and 70 °C.

T (°C)	α	$\beta \;{(10}^{4}\;s)$	%VAR	%MRE
40	1.02 ± 0.03	2.3 ± 0.4	99.5 ± 0.1	9.36 ± 0.33
70	0.86 ± 0.02	1.1 ± 0.4	99.6 ± 0.1	10.14 ± 0.94

Weibull parameters (α and β) and statistical parameters (%VAR and %MRE). For Weibull parameters and %MRE, average values ± LSD intervals are given (n = 3).

**Table 2:** Chemical composition of porcine liver dried at 40 °C (D-40 °C) and 70 °C (D-70 °C) and subsequently defatted (DD-40 °C and DD-70 °C).

Sample	Moisture (%)	Protein (%)	Fat (%)	Ash (%)
D-40 °C	11.24 ± 0.40 A	61.10 ± 1.00 C	22.77 ± 1.57 A	3.99 ± 0.14 B
D-70 °C	7.86 ± 0.27 B	66.53 ± 0.06 B	19.60 ± 0.55 B	4.32 ± 0.10 B
DD-40 °C	8.60 ± 0.22 B	83.67 ± 1.15 A	3.36 ± 0.96 C	4.66 ± 0.88 AB
DD-70 °C	6.62 ± 0.31 C	82.98 ± 1.12 A	5.05 ± 0.05 C	5.39 ± 0.45 A

%: chemical composition (moisture, protein, fat, and ash) g/100 g product. Average values ± LSD intervals are given (n = 2). Different capital letters (A, B and C) show homogeneous groups established from LSD intervals (*p* < 0.05) for moisture, protein, fat, and ash content.

**Table 3:** CIE L*a*b* color parameters, chroma (C*) and hue (H°) of porcine liver dried at 40 °C (D-40 °C) and 70 °C (D-70 °C) and subsequently defatted (DD-40 °C and DD-70 °C).

Sample	L* (Lightness)	a* (Redness)	b* (Yellowness)	C* (CHROMA)	H˚ (Hue)
D-40 °C	50.49 ± 0.52 A	8.83 ± 0.48 A	17.50 ± 0.46 A	19.60 ± 0.59 A	63.25 ± 0.91 A
D-70 °C	53.95 ± 1.37 B	7.09 ± 0.29 B	17.06 ± 0.54 AB	18.47 ± 0.61 A	67.44 ± 0.28 B
DD-40 °C	73.95 ± 0.48 C	4.39 ± 0.08 C	16.35 ± 0.06 B	16.93 ± 0.04 B	74.97 ± 0.31 C
DD-70 °C	67.99 ± 0.53 D	4.97 ± 0.09 D	16.94 ± 0.04 B	17.66 ± 0.03 C	73.66 ± 0.28 D

Average values ± LSD intervals are given (n = 3). Different capital letters (A, B, C and D) show homogeneous groups established from LSD intervals (*p* < 0.05) for lightness, redness, yellowness, chroma, and hue.

**Table 4:** DSC parameters (enthalpy of denaturation: ΔH; initial temperature: Ti; denaturation points: T_d1_ and T_d2_) of porcine liver samples dried at 40 °C (D-40 °C) and 70° C (D-70 °C), and subsequently defatted (DD-40 °C and DD-70 °C), reconstituted at 19.2% protein (*w*/*v*), as raw porcine liver.

Sample	ΔH_total_ (J/g)	T_i_ (°C)	T_d1_ (°C)	T_d2_ (°C)
D-40 °C	1.25	49.16	65.87	85.10
D-70 °C	0.54	53.45	66.26	85.30
DD-40 °C	1.34	50.31	66.39	-
DD-70 °C	0.46	48.65	63.92	86.84
Raw porcine liver	3.46	52.29	66.44	86.90

**Table 5:** Soluble protein (%) and protein solubility (soluble protein/total protein, %) of porcine liver dried at 40 °C (D-40 °C) and 70 °C (D-70 °C) and subsequently defatted (DD-40 °C and DD-70 °C).

Sample	Soluble Protein (%)	Solubility (%)
D-40 °C	27.95 ± 0.31	45.74 ± 0.50
D-70 °C	12.43 ± 0.31	18.69 ± 0.46
DD-40 °C	35.63 ± 2.26	42.60 ± 2.91
DD-70 °C	14.64 ± 0.09	17.64 ± 0.30

Average values ± LSD intervals are given (n = 2).

**Table 6:** Surface functional properties (foaming capacity; foam stability: RFS; emulsifying activity: EAI; emulsion stability: ESI) of porcine liver dried at 40 °C (D-40 °C) and 70 °C (D-70 °C) and subsequently defatted (DD-40 °C and DD-70 °C), compared to pork heart protein and pork spleen protein (pH 6.5).

Sample	Foaming Capacity (mL)	Foam Stability (RFS) (min)	Emulsifying Activity (EAI) (m^2^/g)	Emulsion Stability (ESI) (min)
D-40 °C	235.62 ± 34.2 _B_	7.43	74.85 ± 32.61 _A_	26.44 ± 0.42 _B_
D-70 °C	81.16 ± 4.53 _A_	27.79	92.51 ± 14.43 _A_	15.12 ± 2.83 _A_
DD-40 °C	700.31 ± 32.35 _D_	13.76	72.01 ± 36.08 _A_	22.56 ± 5.19 _B_
DD-70 °C	403.17 ± 4.53 _C_	7.23	72.01 ± 13.40 _A_	13.39 ± 0.27 _A_
Pork heart protein 1	364.9	5.41	354.74	35.19
Pork spleen protein 2	712.1	25.11	497.3	57.6

Average values ± LSD intervals are given (n = 3). Different capital letters (A, B, C and D) show homogeneous groups established from LSD intervals (*p* < 0.05) for foaming capacity, foam stability, emulsifying activity, and emulsion stability. (1) Parés et al. [35]; (2) Toldrà et al. [29]).

## Data Availability

The data presented in this study are available on request from the corresponding author.
